# Whole Genome Sequencing demonstrates that Geographic Variation of *Escherichia coli* O157 Genotypes Dominates Host Association

**DOI:** 10.1038/srep14145

**Published:** 2015-10-07

**Authors:** Norval J. C. Strachan, Ovidiu Rotariu, Bruno Lopes, Marion MacRae, Susan Fairley, Chad Laing, Victor Gannon, Lesley J. Allison, Mary F. Hanson, Tim Dallman, Philip Ashton, Eelco Franz, Angela H. A. M. van Hoek, Nigel P. French, Tessy George, Patrick J. Biggs, Ken J. Forbes

**Affiliations:** 1 School of Biological Sciences, The University of Aberdeen, Cruickshank Building. St Machar Drive, Aberdeen, Scotland, United Kingdom, AB24 3UU; 2 School of Medicine and Dentistry, The University of Aberdeen, Foresterhill, Aberdeen, Scotland, United Kingdom, AB25 2ZD; 3 Centre for Genome Enabled Biology, 23 St Machar Drive, Aberdeen, Scotland, United Kingdom, AB24 3UU; 4 Laboratory for Foodborne Zoonoses, Public Health Agency of Canada, 225089 Township Road 9-1 (Box 640), Lethbridge, Alberta, Canada, T1J 3Z4; 5 Scottish E. coli O157/VTEC Reference Laboratory, NHS Lothian, Royal Infirmary of Edinburgh, 51 Little France Crescent, Edinburgh, Scotland, United Kingdom, EH16 4SA; 6 Public Health England, 61 Colindale Avenue, London, United Kingdom, NW9 5EQ; 7 National Institute for Public Health and the Environment (RIVM), Centre for Infectious Disease Control (CIb), P.O. Box 1, 3720 BA Bilthoven, the Netherlands; 8 mEpiLab, Infectious Disease Research Centre, Institute of Veterinary Animal and Biomedical Sciences, Massey University, Palmerston North, New Zealand

## Abstract

Genetic variation in an infectious disease pathogen can be driven by ecological niche dissimilarities arising from different host species and different geographical locations. Whole genome sequencing was used to compare *E. coli* O157 isolates from host reservoirs (cattle and sheep) from Scotland and to compare genetic variation of isolates (human, animal, environmental/food) obtained from Scotland, New Zealand, Netherlands, Canada and the USA. Nei’s genetic distance calculated from core genome single nucleotide polymorphisms (SNPs) demonstrated that the animal isolates were from the same population. Investigation of the Shiga toxin bacteriophage and their insertion sites (SBI typing) revealed that cattle and sheep isolates had statistically indistinguishable rarefaction profiles, diversity and genotypes. In contrast, isolates from different countries exhibited significant differences in Nei’s genetic distance and SBI typing. Hence, after successful international transmission, which has occurred on multiple occasions, local genetic variation occurs, resulting in a global patchwork of continental and trans-continental phylogeographic clades. These findings are important for three reasons: first, understanding transmission and evolution of infectious diseases associated with multiple host reservoirs and multi-geographic locations; second, highlighting the relevance of the sheep reservoir when considering farm based interventions; and third, improving our understanding of why human disease incidence varies across the world.

Most (61%) emerging infectious diseases are of zoonotic origin and spill over from animal reservoirs to humans, followed to a lesser or greater extent by cycling in the human population^1^. Drivers of genetic variation play a major role in the emergence and evolution of these pathogens^2^. These drivers, acting through selection, include factors that are associated with the relationship between pathogen and host leading to host association as well as geographical separation^3^.

*E. coli* O157 is a relatively rare human pathogen, but sequelae can be severe. The incidence of human *E. coli* O157 infection is 0.51, 0.98, 3.3 and 4.1 cases per 100,000 population in the Netherlands^4^, USA^5^, New Zealand^6^ and Scotland^7^ respectively. Human disease symptoms include bloody diarrhoea in ~90% of cases which can result in haemorrhagic colitis (HC) with 10–15% of cases progressing to haemolytic uraemic syndrome (HUS) and occasionally death^8^.

Cattle and sheep are regarded as the natural asymptomatic reservoirs of *E. coli* O157^9^ which can also be found intermittently in other animals including goats, deer, pigs, wild birds and invertebrates^10^. The scientific literature provides twelve times as many references on *E. coli* O157 and cattle (n = 7,729) compared with sheep (n = 649) (Source: Web of Knowledge, 2014). However, sheep shed similar loads of *E. coli* O157 into the environment as cattle^11^ and are also linked with outbreaks of human infection (e.g. in Scotland for every 3 outbreaks associated with cattle one was associated with sheep^12^). There have been no published studies that have investigated the genetic variation of *E. coli* O157 obtained from cattle and sheep populations at a genomic level and as such the extent of host association is unclear. There is also a need to determine the relative importance of infectious disease reservoirs in order to inform the selection of interventions to reduce infection^13^.

The *E. coli* O157 pathogen was first recognised in 1982^14^ and has since been reported in all continents except Antarctica^15^. Transmission between countries has been hypothesised to be due to animal movements (e.g. transport of cattle and sheep or bird migration) and/or transport of contaminated feed^16^. It has also been found that cattle and human clinical isolates are considerably different between the USA and The Netherlands^17^ whilst Locus Specific Polymorphism Analysis (LSPA6) genotyping and analysis of Shiga toxin bacteriophage insertion (SBI) sites differentiated between isolates from the USA, Argentina and Australia^18^. However, it is unclear whether genetic differences caused by geographic separation of *E. coli* O157 are more pronounced than those caused by host specificity in its primary reservoirs (i.e. cattle and sheep).

Recent developments in next generation sequencing now make it possible to routinely whole genome sequence (WGS) bacterial pathogens and utilise single nucleotide polymorphisms (SNPs) to characterise isolates^19^,^20^. This has proven useful to identify clades of differing virulence and to differentiate between isolates from cattle and clinically ill people^21^,^22^.

Here, WGS is used to determine the extent of host association between cattle and sheep isolates originating from NE Scotland and compares this with clinical isolates obtained from the human population and from food and the environment. These Scottish isolates are then compared with a group of international isolates to determine whether geographic separation is more important than host association as a driver for population differentiation.

## Results

### Analysis of Scottish Isolates

All 145 isolates provided positive in silico PCR results for *rfb* (O-antigen-encoding region of O157), *eaeA* (intimin) and *hlyA* (enterohemolysin) confirming they were *E. coli* O157 and had these putative virulence factors present (Table 1).

**Figure 1:**
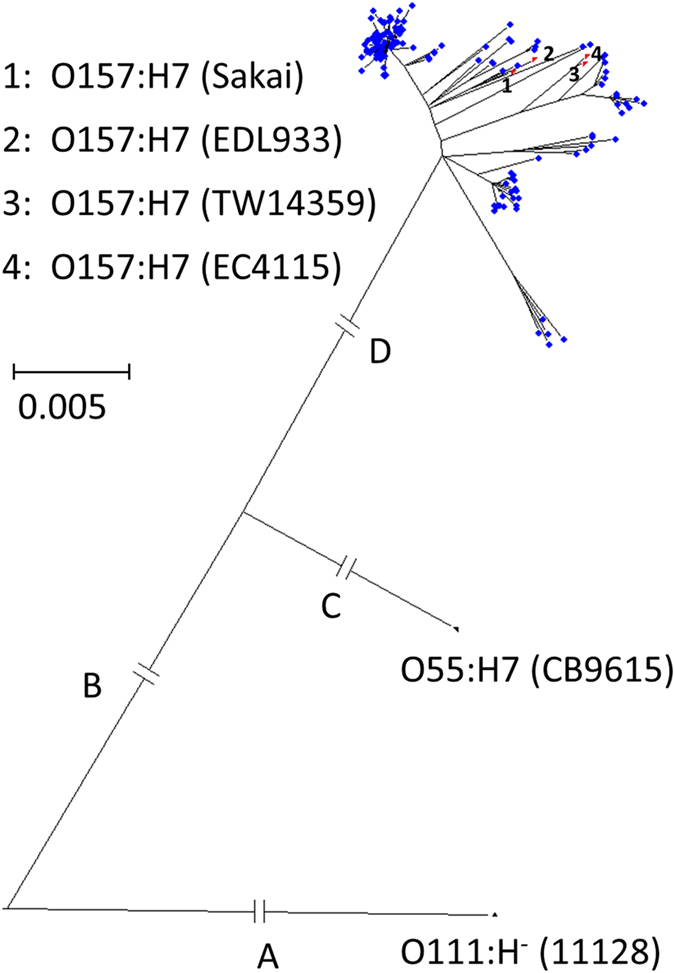
Phylogenetic tree of 145 Scottish *E. coli* O157:H7 isolates. (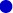), 4 reference USA *E. coli* O157 isolates (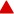) and *E. coli* O55:H7 (CB9615) and *E. coli* O111:H- (11128). The branches A & B are scaled down by a factor of 5 and C & D by a factor of 2.

**Figure 2:**
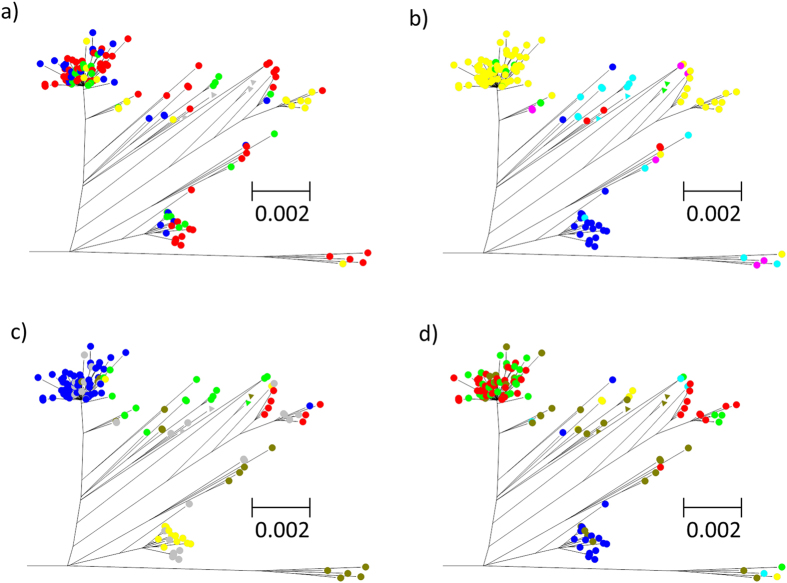
Phylogenetic trees of 145 Scottish *E. coli* O157:H7 isolates and 4 reference USA isolates. Illustrating (**a**) Sources: Clinical (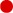); Cattle (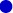); Sheep (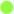); Food/Environment (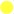); Unknown (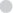); International (▵), (**b**) Shiga toxins: *stx1a/stx2a* (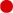); *stx1a/stx2c* (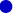); *stx2a* (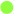); *stx2a/stx2c* (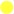); *stx2c* (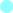); *negative* (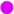); Unknown (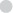); International (▵), (**c**) Phage types: PT2 (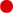); PT21/28 (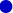); PT32 (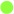); PT8 (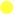); Other PTs (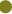); Unknown (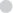); International (▵) and (**d**) SBI genotypes: ASY2a2c (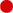); SY1a2c (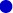); SY2a2c (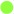); SY2c (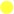); YN (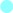); Other SBI types (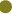); and International (▵).

A phylogenetic tree of the *E. coli* O157 isolates was generated utilising the 8559 SNPs, of which 871 were phylogentically informative, from the core genome (1.223 Mb) generated by PanSeq (Fig. 1, Fig. S1 and Table S2). The tree visualises the radiation of *E. coli* O157:H7 and is parsimonious with the theory of the evolution of *E. coli* O157:H7 from O55:H7. Figure 2(a) shows the distribution of the sources (clinical, cattle, sheep, food/environment) of the isolates across the *E.coli* O157:H7 portion of the phylogeny and it can readily be observed that a number of these sources are present on the same branches of the tree. However, Nei’s genetic distance (Table S6), which provides a measure of the relative distribution of sources across the tree, is significantly different (P < 0.05) between each pair of sources except for cattle and sheep (P > 0.05).

The most common Shiga toxin subtypes in the Scottish isolates (Table 1) were *stx2a*/*stx2c* (62%) and *stx1a*/*stx2c* (15%). The distribution of the Shiga toxin subtypes did show some association with source (*χ*^2^ = 26.4, df = 15, P = 0.03). Fisher’s exact test indicated a preponderance of the *stx1a*/*stx2c* subtype in bovine isolates compared to those from food and the environment and also that the *stx2c* subtype was more prevalent in ovine than clinical and food/environmental isolates. Figure 2(b) shows clusters of Shiga toxin subtypes in the *E. coli* O157 phylogenetic tree confirmed by Nei’s genetic distance (Table S6).

The majority (53%) of isolates from all sources were PT21/28 (Table 1). The distribution of phage types by source was found to be indistinguishable (*χ*^2^ = 10.0, df = 12, P > 0.05). Phage types appeared to cluster on the phylogenetic tree (Fig. 2(c)) and significant genetic distances were found between all pairwise combinations of phage types (Table S6).

The LSPA6 lineage I/II subtype 211111 was present in 94% of the Scottish isolates and was also found in >90% in each source (Table S5), whilst only 5 Scottish isolates (4 clinical and 1 Food/Environment) were *tir* 255 allele A. In total 14 Scottish isolates were Manning clade 8, 2 were clade 3 and the remaining 129 clades 4/5/6/7/9 (Table S5).

**Figure 3:**
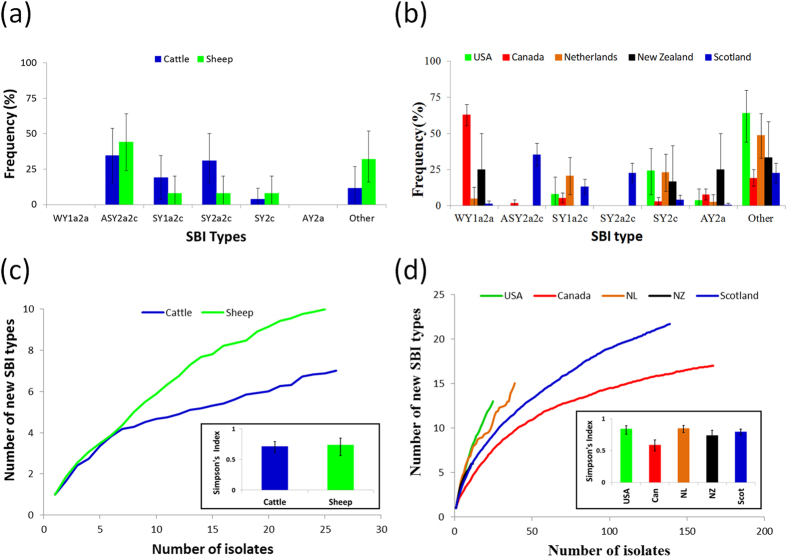
Variation of Shiga toxin Bacteriophage Insertion (SBI) types between ruminant hosts and between countries. Frequency of predominant SBI types (**a,b**), rarefaction and Simpson’s diversity index (**c**,**d**).

The three most common SBI genotypes ASY2a2c (35.2%), SY1a2c (13.1%) and SY2a2c (22.8%) comprised approximately 60% of all isolates (Table 1). However, the remaining isolates exhibited a considerable diversity of SBI types (n = 24). Overall there were no significant differences in the distribution of SBI genotypes by source (*χ*^2^ = 19.6, df = 15, P = 0.19). Fisher’s exact test utilising pairwise comparisons from ovine and bovine isolates also showed that the main SBI genotypes were in similar proportions (Table 1 and Fig. 3(a)). When SBI genotype was plotted on the phylogenetic tree (Fig. 2(d)) it appears that there is clustering between all the main genotypes which was confirmed by Nei’s genetic distance (P < 0.05, Table S6).

Although rarefaction appears to show sheep have a greater diversity of SBI types than cattle (Fig. 3(c)) this was not significant (Table S7). Simpson’s index (Fig. 3(c)) also found no difference in diversity between Scottish cattle and sheep sources (Table S7). Isolates from food were found to have the most significant differences in SBI genotypes when compared pairwise with other sources (6 of the 8 significant differences in Table 1).

### Analysis of International isolates

Of the 429 genomes 6, 9 and 20 gave negative in silico PCR results for *rfb* (O-antigen-encoding region of O157:H7), *eaeA* (intimin) and *hlyA* (enterohemolysin) respectively (Table S1). Of the 6 that were negative for *rfb*_O157_, 2 were positive for a single primer and all 6 were positive for a SNP in *uid*A which is an indicative marker for *E. coli* O157^23^. The Shiga toxin types, LSPA6 genotypes, SBI types, *tir* 255 A/T variants and Manning clade allocation are given in Table S1.

There were significant differences (*χ*^2^ = 127.0, df = 24, P < 0.0001) in the distribution of SBI types by country (Fig. 3(b)) with an excess of type WY1a2a from Canada and ASY2a2c and SY2a2c of Scottish origin. Although rarefaction (Fig. 3(d)) appears to show a lower diversity of Canadian SBI types compared with other countries this was not found to be statistically significant (Table S7). Whereas Simpson’s diversity index (Fig. 3(d) and Table S7) showed statistically significant differences in diversity amongst a number of countries but with Canada having the lowest diversity of all.

**Figure 4:**
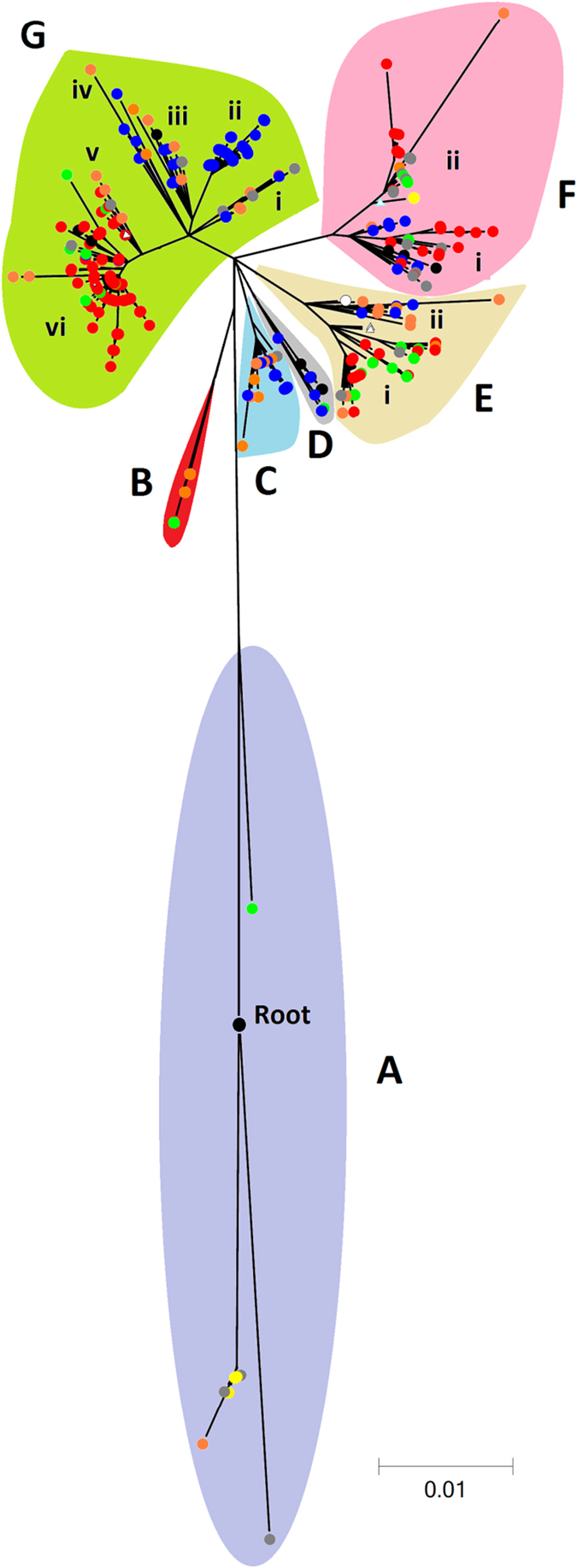
Phylogenetic tree of international isolates. Scotand (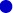); Canada (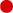); USA (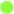); The Netherlands (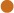); New Zealand (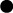); Europe (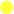); Egypt (○); Asia (▵); South America (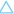); Unknown (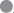). Letters A–G denote the phylogenetic clades identified.

In total, 813 SNPs were obtained from PanSeq from a core genome of 827 kb (Table S3). The resulting bootstrapped concensus phylogeny was broken into clades (Fig. 4, Figs S3 & S4). Clade A is ancestral from which the root to *E. coli* O55:H7 is located (Fig. 4). This clade comprises isolates from USA, the Netherlands and other European countries. Clade B comprises isolates from the Netherlands and America. Clades C-G all branch from the same point with sub-clade structure being apparent in clades E, F and G. Clades C and E(ii) are European, E(i) being predominantly North American and Dutch and D comprising New Zealand and Scottish isolates. Clade F is formed from a number of different countries with a lack of Scottish isolates being apparent in F(ii). Clades G(i) – G(iv) are primarily European with G(ii) Scottish and G(iii) Scottish, Dutch and New Zealand isolates. Clades G(v) and G(vi) are mainly North American, with some Dutch and Asian isolates. Nei’s genetic distance utilising the 813 SNPs demonstrated significant inter-country differences (Table S9) which is in agreement with the geographical structuring of isolates on the phylogenetic tree. This phylogeny also appears to be parsimonious with previous typing approaches (Fig. S4).

## Discussion

The finding that an indistinguishable population of *E. coli* O157 is circulating through both cattle and sheep agrees with results from Sweden that used MLVA typing^24^. This is of importance to the many countries (e.g. UK, New Zealand etc.) that have significant populations of both cattle and sheep as there is opportunity for transmission between species when they are located on the same farm, co-grazing or at livestock markets. On farm intervention strategies (e.g. feeding probiotics, providing clean bedding etc.^25^) targeted at cattle may be hampered by sheep reseeding *E. coli* O157 into the agricultural environment. Hence, there is a need to develop and apply multi-species transmission network models for this pathogen^26^. The carriage of *E. coli* O157 by both cattle and sheep appears not to increase the genetic diversity of this pathogen perhaps because the ecological niche is similar within these hosts.

Although it appears that *E. coli* O157 genotypes exhibit a generalist behaviour within ruminants it is unknown whether all of these genotypes have sufficient phenotypic plasticity to be found in a range of other animal hosts^9^. Other gastrointestinal pathogens (e.g. *Campylobacter* and *Salmonella*) have a number of specialist subtypes associated with particular animal host species^27^,^28^, but it is yet to be established whether this is also the case for *E. coli* O157.

The international phylogeographic tree of *E. coli* O157 (Fig. 4) shows geographical structuring. There appears to be greater isolation exhibited by the Scottish and North American isolates. Whereas, both the Dutch and New Zealand isolates cluster on occasions most closely with those from Scotland and on other occasions with those from North America. This is suggestive of multiple transmission events between New Zealand, Europe and North America followed by genetic radiation of isolates within a country. This geographic structuring agrees with previous PFGE and sequence based studies and sequence based analysis^6^,^16^,^18^.

The question arises as to how and when these transmission events may have occurred. There is documented evidence of intermittent trade comprising relatively low numbers of live cattle and sheep, predominantly for breeding purposes, between the UK, USA, Canada, New Zealand and the Netherlands for the past 20 years (www.uktradeinfo.com). Prior to this international transport of live animals certainly occurred^29^ and may have taken place via intermediate countries (e.g. UK to Australia to New Zealand). *E. coli* O157 prevalence in cattle/sheep is approximately 5%^30^, and shedding within a cattle herd can last >5 months^31^. This time is sufficient for the organism to survive the sea crossing (50 days) between the UK and New Zealand. Transmission via contaminated feed/food exported between countries is also plausible because of the complex transportation networks^32^. Carriage of *E. coli* O157 by humans between countries can occur^33^, but secondary transmission between humans is low^34^ and transmission to cattle or sheep is also likely to be low because of the general effectiveness of sewerage systems. It seems unlikely that direct transmission by wild animals and migrating birds between continents takes place because of the intermittent nature of carriage in these hosts but certainly over shorter distances (e.g. between adjacent regions) transmission is plausible^35^.

From a public health perspective there is considerable interest in elucidating why there is heterogeneity in the *E. coli* O157 clinical disease burden between countries. Exposure is of importance with foodborne routes being dependent on local food preferences and patterns of food distribution^10^ and environmental routes being dependent on exposure to cattle/sheep faeces and contaminated water. Susceptibility is also of importance with young children being at particular risk^36^. However, establishment of *E. coli* O157 in the ruminant population within a country is initially dependent on inward transmission and subsequent reservoir competence. Further, since humans are effectively an evolutionary dead end for *E. coli* O157^10^, its pathogenicity in humans is an accidental consequence of its ecology within the extant agricultural system. Hence, the reason why human disease incidence varies between continents, countries and even states/regions is likely to be multifactorial. NE Scotland has a high disease burden, and a large ruminant population which offers the opportunity of environmental and foodborne exposure. Characteristics of the pathogen are also important with PT21/28 isolates (clade G(ii)) dominating, which is known to be excreted at high levels by cattle^37^ and carries the most potent Shiga toxin gene *stx2a*^38^.

Comparisons between cattle sources and humans within countries (Scotland, USA, Netherlands and Canada) also showed significant differences (Table S9). This was not found to be the case for New Zealand here, possibly because sample size was small, but previous studies observed differences when investigating the distribution of SBI types both in the USA^39^ and New Zealand^6^. These results indicate that there is a different population structure of *E*. *coli* O157 between cattle and humans in each country. It is plausible that some isolates from cattle are more likely than others to cause infection in humans as reported elsewhere^17^. It can be concluded here that human cases arise from all of the clades and sub-clades across the tree and that in Clade F most isolates have the potent *stx2a* toxin (this agrees with the hypothesis of Manning clade 8 being virulent^21^) whilst Clades C, D and E have a very low occurrence of *stx2a* which suggests that human disease arising from these clades is likely to be less severe.

The aim of this work was not to identify the origin of *E. coli* O157 from its *E. coli* O55:H7 progenitor but the Clade A isolates closest to the *E. coli* O55:H7 root of the international tree (Fig. 4) originate from North America and Europe suggesting that inter-continental spread of this organism occurred fairly early in its evolutionary origins. This is corroborated by a survey of isolates deposited in culture collections prior to 1982 which noted 7 from North America and 1 from the UK^40^.

This work has also been able to illustrate how part of the dynamic accessory genome (e.g. Shiga toxin encoding phage) relate to the evolutionary core genome of this pathogen. There remains the vast majority of the accessory genome to explore which may contain genes for virulence and/or adaptive advantage in specific niches^19^. The comparisons of the international SNP based phylogeny with existing typing techniques (e.g. LSPA6 typing, *tir* A/T, Manning clades etc see Fig. S4 and Table S5 & S8) shows the backward compatibility of the SNP based methodology with existing methods.

## Conclusion

*E. coli* O157 continues to be a pathogen that causes significant levels of disease and morbidity across the world. It transmits freely between its primary reservoirs of cattle and sheep and there is a lack of evidence of genetic differences between the populations of isolates originating from these hosts. It is therefore important to consider the sheep reservoir when investigating interventions to reduce the presence of this pathogen, particularly at the farm level. The pathogen appears to have been transmitted a number of times between countries and continents but the rate of transmission is low enough that a strong phylogeographic signal remains. Achieving a global understanding of this pathogen through whole genome sequencing is important in terms of understanding the potential origins of outbreaks at national and continental spatial scales, evolution of this pathogen and the relative importance of its animal reservoirs and subsequent pathogenicity to humans.

## Materials and Methods

### Isolates

A total of 145 Scottish isolates (Table S1), comprising clinical isolates (74) originating from patients resident in NE Scotland, cattle (26), sheep (25) and food/environmental (20) isolates from previous studies. Isolates were selected to be independent of each other (i.e. not to be obtained from the same farm at the same time) and to be obtained over a broad time period. Isolates were phage typed as described previously^41^.

### Sequenced isolates

Sequenced international *E. coli* O157 isolates from Canada (164), Netherlands (39), New Zealand (12), Scotland (145), USA (26) and other countries (45) were obtained along with varying levels of metadata (Table S1). The Canadian and Dutch sequenced isolated were selected using the same principles as the Scottish isolates, the New Zealand isolates were obtained from two calendar years but variation was ensured by selection across their PFGE profiles. The genomic sequence data of these isolates will be uploaded to an open access database on acceptance of this paper for publication (Table S1).The isolates from USA and the rest of the world were downloaded from the NCBI and PATRIC databases.

### Whole genome sequencing of Scottish isolates

Overnight cultures of *E. coli* O157 were grown on Harlequin SMAC-BCIG agar plates (Hal 6, Lab M, Topley House, Lancashire) at 37 °C. A single, well isolated sorbitol negative colony was selected and tested with *E. coli* O157 latex (code DR0620M, Oxoid, Basingstoke) and the latex plated onto Columbia Agar at 37 °C for 24 hours (Code CM0031, Oxoid, Basingstoke). DNA was extracted with the Wizard Genomic DNA Purification Kit (Promega UK Ltd, Southampton) as per the manufacturers instructions with an additional Proteinase K step (25 μl, lyophilized Proteinase K reconstituted at a concentration of 6 mg/260 μl Proteinase buffer) post treatment with nucleic acid lysis solution. The concentration of DNA was determined by Picogreen assay and then submitted to the Wellcome Trust Centre for Human Genetics (WTCHG), Oxford for WGS. This was conducted using an Illumina HiSeq sequencer with 100 base paired-end sequencing. The FASTQ paired-end reads were assembled using Velvet^42^, coverage was typically 30x and assembled genome size approximately 5.5 Mb.

### Analysis of genomes

In silico PCR and probe based assays (Table S4) were carried out both for backwards compatibility with previous studies and identification of known virulence markers. SNP discovery methods were also implemented for phylogenetic analysis.

1. *Detection of E. coli* O*157 antigen encoding, intimin and enterohemolysin genes*: in silico PCRs (Table S4), performed using a Perl script were conducted for a portion of the *rfb* (O-antigen-encoding) region of *E. coli* serotype O157 and for the putative accessory virulence factors intimin (*eaeA*) and the plasmid-encoded enterohemolysin (*hlyA*). The Perl script blast searched for the primers in the genome sequences with tolerance of 90%. If the pair of primers were found, the size of the PCR product was determined and if matching the predicted size the PCR was assigned as positive.
2. *Shiga toxin subtyping:* an in silico combined BLAST and SNP based approach was employed to detect the presence of Shiga toxin genes (Table S4). This allows for the homology between the different Shiga toxin types and compares the FASTQ reads and/or genome assemblies with reference Shiga toxin genes^43^. Odds ratios (OR) were calculated to determine whether particular Shiga toxin types were more or less frequent in particular sources (cattle, sheep, clinical and food/environment). P values were obtained from a two-tailed Fisher’s exact test.
3. *LSPA6 sub-typing, tir 255T* > *A polymorphism analysis and Clade Identification (Manning):* was performed in silico (Table S4).
4. *SBI sub-typing:* in silico PCRs were performed to determine prophage occupancy of *E. coli* O157 loci *argW*, *sbcB*, *wrbA*, *yehV* and SBI results were concatenated using the terminology described previously^44^ (see Table S4).
5. *Rarefaction:* was used to compare the diversity and distribution of SBI types between animal host species and between countries and testing for significance was carried out using a randomization test^45^ implemented with Poptools (http://www.cse.csiro.au/poptools/; accessed 2014 June 23).
6. *Simpson’s Index:* was used as a measure of the taxonomic richness of SBI types from different host species or different countries^46^. An index value of 0 indicates homogenous types and a value of 1 indicates a totally heterogeneous population. Testing for significance was carried out using a randomization test as above.
7. *Pan-genomic SNP analysis:* two analyses were performed. The first comprised the 145 Scottish genomes together with four reference *E. coli* O157:H7 published genomic sequences (TW14359 and EC4115 from the USA spinach outbreak^47^, EDL933 and Sakai), along with an *E. coli* O55:H7 (CB9615) and an *E. coli* O111:H- (11128). The second comprised the Scottish and all of the international *E. coli* O157 genomes in the study. PanSeq was used to construct a non-redundant pan-genome for each of the two analyses^20^. The pan-genome was constructed by using a seed genome and identifying regions of ≥1000 bp not found in the seed but present in any other genome at a 99 percent sequence identity cutoff. The pan-genome was subsequently fragmented into 1000 bp segments, and the presence/absence of each locus in every genome determined at a 99 percent sequence identity threshold. Loci present in all genomes underwent multiple sequence alignment using Muscle^48^, and were concatenated together. This aligned pan-genome was used to identify SNPs in the core genome of all isolates. A phylogeny of *E. coli* O157 isolates’ genomes was rooted using the *E. coli* O55:H7 strain CB9615 as a proximal outgroup (O55:H7 is considered to be the immediate ancestor of *E. coli* O157), and *E. coli* O111:H- strain 11128 as a more distal outgroup. A neighbour joining tree was generated and visualised in MEGA^49^. Bootstrapping with 500 iterations was also performed and concensus trees generated.
8. *Genetic distance*: Nei’s standardized genetic distance *d*_1_, was calculated for each pairwise combination of sources (e.g. cattle and sheep), phage types, Shiga toxin types, LSPA6 lineages, SBI genotypes and countries^50^ utilising the pan-genomic SNP data detailed above. The genetic distance ranges from 1 (where there are no SNPs in common between the two groups) and 0 (where the two groups have an identical distribution of SNPs).

## Additional Information

**How to cite this article**: Strachan, N. J. C. *et al.* Whole Genome Sequencing demonstrates that Geographic Variation of *Escherichia coli* O157 Genotypes Dominates Host Association. *Sci. Rep.* **5**, 14145; doi: 10.1038/srep14145 (2015).

## Supplementary Material

Supplementary Information

Supplementary Table S1

Supplementary Table S2

Supplementary Table S3

## Figures and Tables

**Table 1:** Abundance of genotype/phenotype factors by source (Bovine, Ovine, Food/Environment and Clinical) for the Scottish *E. coli* O157 isolates.

Factor	Sub-type	No. (%) of strains with factor^a^				
		Bovine(n = 26)	Ovine(n = 25)	Food/Env.(n = 20)	Clinical(n = 74)	Statistically significantpairwise comparisons^b^
O encoding antigen	*rfb*_O157_	26 (100.0)	25 (100.0)	20 (100.0)	74 (100.0)	NA
Intimin	*eae*	26 (100.0)	25 (100.0)	20 (100.0)	74 (100.0)	NA
Enterohemolysin	*hlyA*	26 (100.0)	25 (100.0)	20 (100.0)	74 (100.0)	NA
Shiga toxin	*stx1a*/*stx2a*	0 (0.0)	0 (0.0)	1 (5.0)	3 (4.1)	none
	*stx1a/stx2c*	6 (23.1)	4 (16.0)	0 (0.0)	12 (16.2)	BF
	*stx2a*	0 (0.0)	0 (0.0)	3 (15.0)	5 (6.8)	none
	*stx2a/stx2c*	18 (69.2)	14 (56.0)	13 (65.0)	45 (60.8)	none
	*stx2c*	2 (7.7)	6 (24.0)	0 (0.0)	5 (6.8)	CO, FO
	−ve	0 (0.0)	1 (4.0)	3 (15.0)	4 (5.4)	none
Phage Type	PT2	1 (3.8)	1 (4.3)	2 (22.2)	3 (5.8)	none
	PT21/28	16 (61.5)	11 (47.8)	4 (44.4)	27 (51.9)	none
	PT32	2 (7.7)	3 (13.0)	2 (22.2)	8 (15.4)	none
	PT8	5 (19.2)	4 (17.4)	0 (0.0)	5 (9.6)	none
	Other^c^	2 (7.7)	4 (17.4)	1 (11.1)	9 (17.3)	none
SBI	Sub-type					
	ASY2a2c	9 (34.6)	11 (44.0)	4 (20.0)	27 (36.5)	none
	SY1a2c	5 (19.2)	2 (8.0)	0 (0.0)	12 (16.2)	none
	SY2a2c	8 (30.8)	2 (8.0)	9 (45.0)	14 (8.9)	CF, CO, FO
	SY2c	1 (3.8)	2 (8.0)	0 (0.0)	3 (4.1)	CF, FO
	YN	0 (0.0)	1 (4.0)	1 (5.0)	3 (4.1)	none
	Other^d^	3 (11.5)	7 (28.0)	6 (30.0)	15 (20.3)	none

^a^No of strains phage typed: bovine (n = 26), ovine (n = 23), food/env (n = 9) and clinical (n = 52).

^b^Listed are statistically different pairwise comparisons where B = bovine, O = ovine, F = Food/Environmental and C = clinical. Statistical significance determined by Fisher’s exact test.

^c^Other defines all Phage Types present in <5 Isolates.

^d^Other defines all SBI genotypes present in <5 isolates.
