# Supplementary material for: Whole Genome Sequencing demonstrates that Geographic Variation of Escherichia coli O157 Genotypes Dominates Host Association
Source: Sci Rep. 2015 Oct 7;5:14145. doi: 10.1038/srep14145 (PMC4595763; doi:10.1038/srep14145)
Supplement: Supplementary Information [file srep14145-s1.pdf]

SUPPLEMENTARY INFORMATION

Whole Genome Sequencing demonstrates that Geographic Variation of *Escherichia coli* O157 Genotypes Dominates Host Association

Norval J.C. Strachan, Ovidiu Rotariu, Bruno Lopes, Marion MacRae, Susan Fairley, Chad Laing, Victor Gannon, Lesley J. Allison, Mary F. Hanson, Tim Dallman, Philip Ashton, Eelco Franz, Angela H.A.M. van Hoek, Nigel P. French, Tessy George, Patrick J. Biggs, and Ken J. Forbes.

Metadata

The Metadata, as well as the in silico PCR results, assignment of Shiga toxins and SBI types described in Table 1 are in Supplementary Table S1 (Supplementary_Table_S1.xlsx). This is an Excel file which contains a worksheet for each of USA, Canada, Scotland, New Zealand, the Netherlands and other countries.

SNP data

The SNP data for the Scottish phylogenetic tree (Fig. 1 and Fig. 2) is in Supplementary Table S2 (Supplementary_Table_S2.xlsx).

The SNP data for the international phylogenetic tree (Fig. 4) as well as locations in Sakai (NC_002695.1) of discriminatory SNPS for clades A-G and corresponding probes is in Supplementary Table S3 (Supplementary_Table_S3.xlsx).

Current File

In the current file are Suppl. Figs S1 – S4 and Suppl. Tables S4 to S9.

Fig. S1a depicts the bootstrap phylogenetic tree of the 145 Scottish isolates with 4 reference international *E. coli* O157 (Sakai, EDL933, TW14359 and EC4115), an *E. coli* O55:H7 (CB9615) and an *E. coli* O111:H- isolate (See also Fig. 1). The tree was drawn utilising 871 phylogenetically informative SNPs obtained from Panseq. A Neighbor-Joining method was employed [1]. The optimal tree with the sum of branch length = 1.249 is shown. The evolutionary distances were computed using the Maximum Composite Likelihood method [2] and are in the units of the number of base substitutions per site. All positions containing gaps and missing data were eliminated. There were a total of 882 positions in the final dataset. Evolutionary analyses were conducted in MEGA6 [3]. **Fig. S1b** shows the bootstrap consensus tree of this dataset, inferred from 500 replicates taken to represent the evolutionary history of the taxa analyzed [4]. Branches corresponding to partitions reproduced in less than 50% bootstrap replicates are collapsed. The evolutionary distances were computed using the Maximum Composite Likelihood method [2] and are in the units of the number of base substitutions per site.

Fig. S2 illustrates the phylogeny of the above *E. coli* O157 isolates in radiation format where the isolate labels are coloured to represent their LSPA6 lineage, whether their *tir* 255 SNP is A or T, which of Manning's clades they belong to and their clade membership according to Fig. 3 in the current paper.

Fig. S3a depicts the bootstrap phylogenetic tree of the combined international and Scottish *E. coli* O157 isolates (see also Fig. 3). A Neighbor-Joining method was employed [1]. The tree was drawn utilising 813 phylogenetically informative SNPs obtained from Panseq (Suppl. Table S3). The optimal tree with the sum of branch length = 0.7884 is shown. The evolutionary distances were computed using the Maximum Composite Likelihood method [2] and are in the units of the number of base substitutions per site. All positions containing gaps and missing data were eliminated. There were a total of 812 positions in the final dataset. Evolutionary analyses were conducted in MEGA6 [3]. **Fig. S3b** shows the bootstrap consensus tree of this dataset, inferred from 500 replicates taken to represent the evolutionary history of the taxa analyzed [4]. Branches corresponding to partitions reproduced in less than 50% bootstrap replicates are collapsed. The evolutionary distances were computed using the Maximum Composite Likelihood method [2] and are in the units of the number of base substitutions per site.

Fig. S4 shows the tree depicted in Fig. S3 in radiation format and with isolate labels coloured to show sources (clinical cattle, sheep, food/environment or unknown), Manning's Clade membership, LSPA6 lineage, *tir* 255 allele (T or A), phage type, Shiga toxin, SBI genotype and presence of O-antigen-encoding region of O157 (*rfb* O157).

Table S4 provides a list of the oligonucleotide PCR primers, probes or gene accession numbers used in this study along with the relevant references.

Table S5 is a continuation of Table 1 which shows the abundance of LSPA6, *tir* 255, and Manning clades by source (Bovine, Ovine, Food/Environment and Clinical) for the Scottish *E. coli* O157 isolates.

Table S6 provides pairwise comparisons of Nei's genetic distances calculated using the SNP polymorphisms within the Scottish *E. coli* O157 isolates obtained from Panseq. Distances are calculated between sources, phage types, Shiga-toxin types, LSPA6 lineages and SBI genotypes.

Table S7 presents P-values of randomisation tests for pairwise comparisons of (a) rarefaction and (b) Simpson's index by (i) animal source and (b) country.

Table S8 depicts the allocation of phylogenetic clades from the current study (Fig. 4) to those of previous typing methods (Manning clades, LSPA6 lineages, *tir* 255 A/T alleles, phage types, Shiga toxin types and SBI types).

Table S9 provides pairwise comparisons of Nei's genetic distances calculated using the SNP polymorphisms (Suppl. Table S3) from international *E. coli* O157 isolates between and within countries.

References

1. Saitou, N. & Nei, M. The Neighbor-Joining Method - a New Method for Reconstructing Phylogenetic Trees. *Mol. Biol. Evol.* **4**, 406-425 (1987).

2. Tamura, K., Nei, M. & Kumar, S. Prospects for inferring very large phylogenies by using the neighbor-joining method. *Proc. Natl. Acad. Sci. U. S. A.* **101**, 11030-11035 (2004).

3. Tamura, K., Stecher, G., Peterson, D., Filipski, A. & Kumar, S. MEGA6: Molecular Evolutionary Genetics Analysis Version 6.0. *Mol. Biol. Evol.* **30**, 2725-2729 (2013).

4. Felsenstein, J. Confidence-Limits on Phylogenies - an Approach using the Bootstrap. *Evolution* **39**, 783-791 (1985).

5. Paton, A. & Paton, J. Detection and characterization of Shiga toxigenic Escherichia coli by using multiplex PCR assays for stx(1), stx(2), eaeA, enterohemorrhagic E-coli hlyA, rfb(O111), and rfb(O157). *J. Clin. Microbiol.* **36**, 598-602 (1998).

6. Scheutz, F. *et al*. Multicenter evaluation of a sequence-based protocol for subtyping Shiga toxins and standardizing Stx nomenclature. *J. Clin. Microbiol.* **50**, 2951-2963 (2012).

7. Ashton, P. *et al*. Insight into Shiga toxin genes encoded by*Escherichia coli*O157 from whole genome sequencing. *PeerJ PrePrints* **2:e652v1**, 1-22 (2014).

8. Yang, Z. *et al*. Identification of common subpopulations of non-sorbitol-fermenting, beta-glucuronidase-negative*Escherichia coli*O157:H7 from bovine production environments and human clinical samples. *Appl. Environ. Microbiol.* **70**, 6846-6854 (2004).

9. Bono, J. L. *et al*. Association of*Escherichia coli*O157 : H7 tir polymorphisms with human infection. *BMC Infect. Dis.* **7**, 98 (2007).

10. Manning, S. D. *et al*. Variation in virulence among clades of *Escherichia coli*O157 : H7, associated with disease outbreaks. *Proc. Natl. Acad. Sci. U. S. A.* **105**, 4868-4873 (2008).

11. Shringi, S. *et al*. Carriage of stx2a differentiates clinical and bovine-biased strains of*Escherichia coli*O157. *PLoS One* **7**, e51572 (2012).

12. Whitworth, J. *et al*. Diverse Genetic Markers Concordantly Identify Bovine Origin*Escherichia coli*O157 Genotypes Underrepresented in Human Disease. *Appl. Environ. Microbiol.* **76**, 361-365 (2010).

13. Riordan, J. T., Viswanath, S. B., Manning, S. D. & Whittam, T. S. Genetic differentiation of Escherichia coli O157 : H7 clades associated with human disease by real-time PCR. *J. Clin. Microbiol.* **46**, 2070-2073 (2008).

14. Mellor, G. E. *et al*. Multilocus Genotype Analysis of*Escherichia coli*O157 Isolates from Australia and the United States Provides Evidence of Geographic Divergence. *Appl. Environ. Microbiol.* **79**, 5050-5058 (2013).

Scottish Additional Phylogenetic Figures

(a)

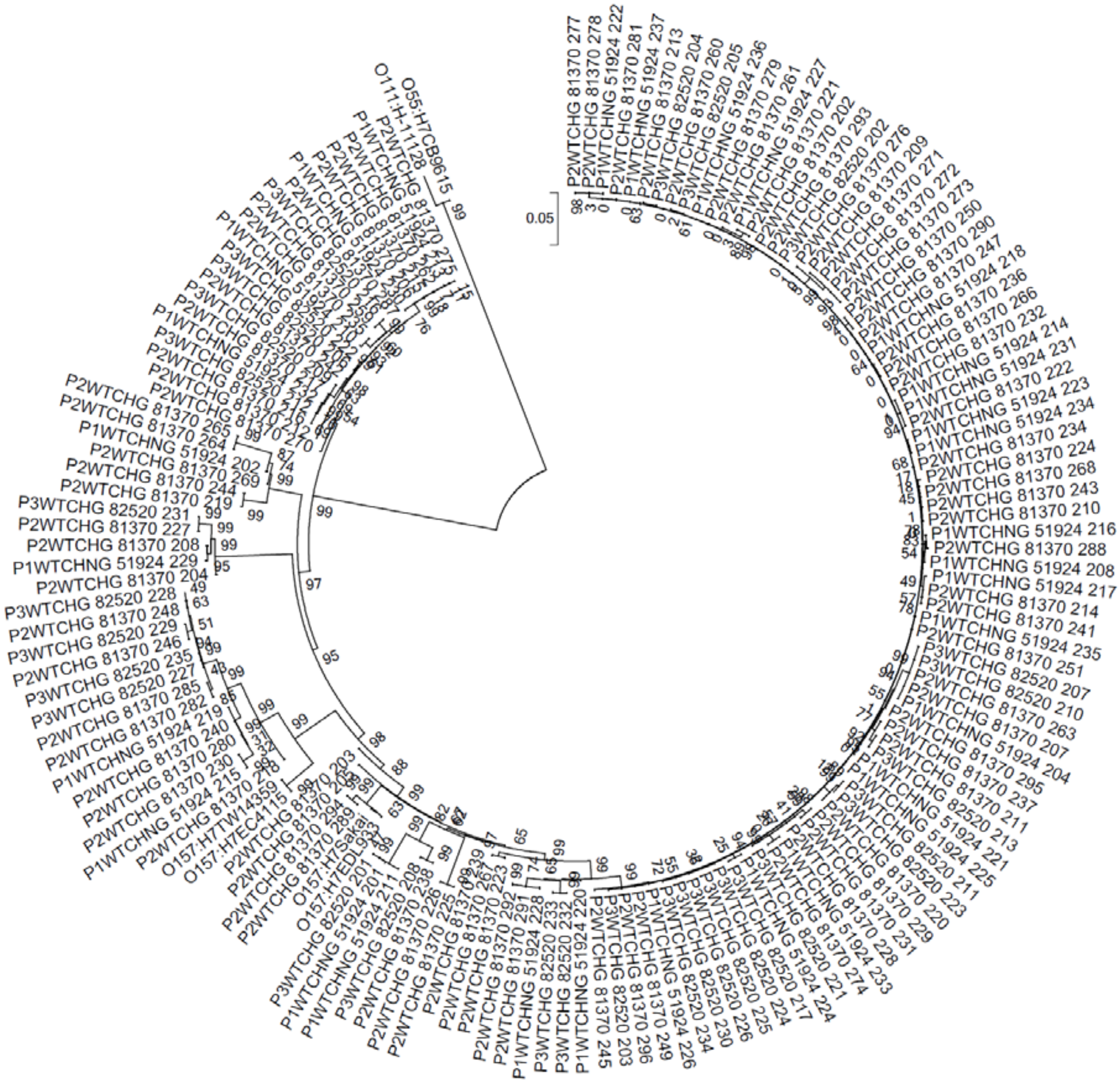

(b)

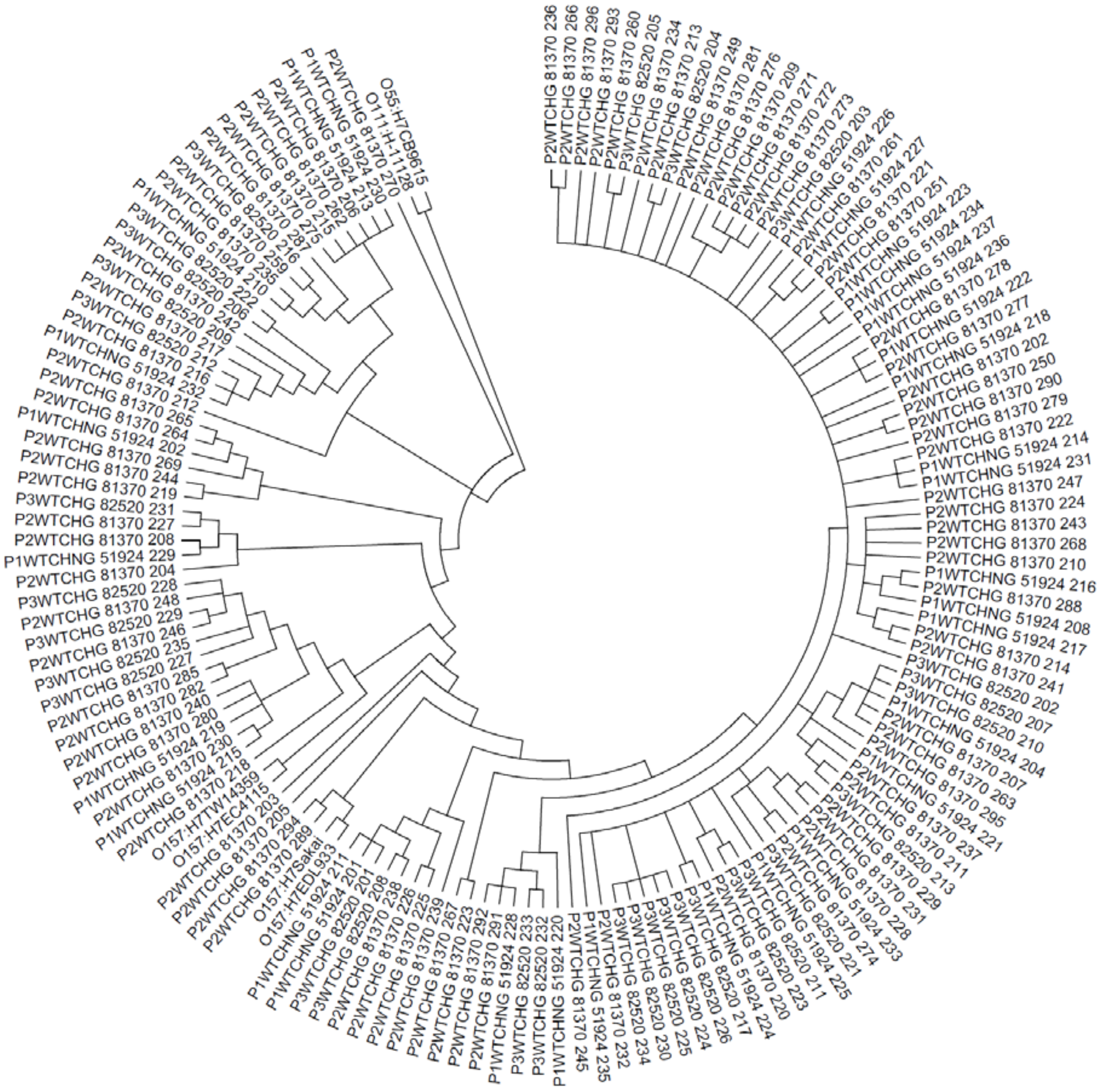

Figure S1 (a) Bootstrapped phylogenetic tree and (b) the Consensus tree for the 145 Scottish isolates, 4 international *E. coli* O157 isolates (Sakai, EDL933, TW14359 and EC4115) and *E. coli* O55:H7 (CB9615) and *E. coli* O111:H-.

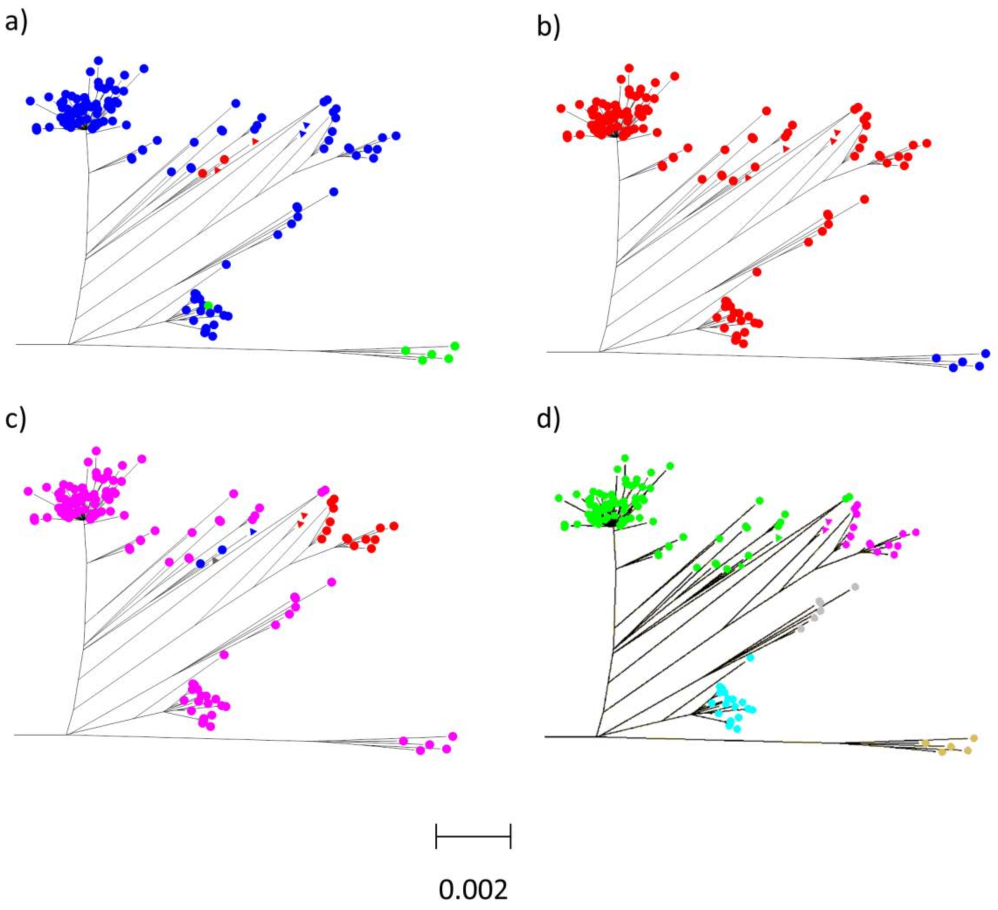

Figure S2 The *E. coli* O157 phylogeny of the 145 Scottish and 4 international isolated described in Figures 1 and 2 characterised by (a) LSPA6 lineage: I (●); I/II (●); II (●); Unknown (●); International (△), (b) *tir* 255 genotypes*: tir* T (●); *tir* A (●); International (△); (c) Manning's Clades: Clade 1 (●); Clade 2 (●); Clade 3 (●);Clade 4/5/6/7/9 (●); Clade 8 (●); International (△) and (d) the clades defined in the international tree (Figure 3): C (●); D (●); E (●), F (●) and G (●).

International additional Phylogenetic Figures

(a)

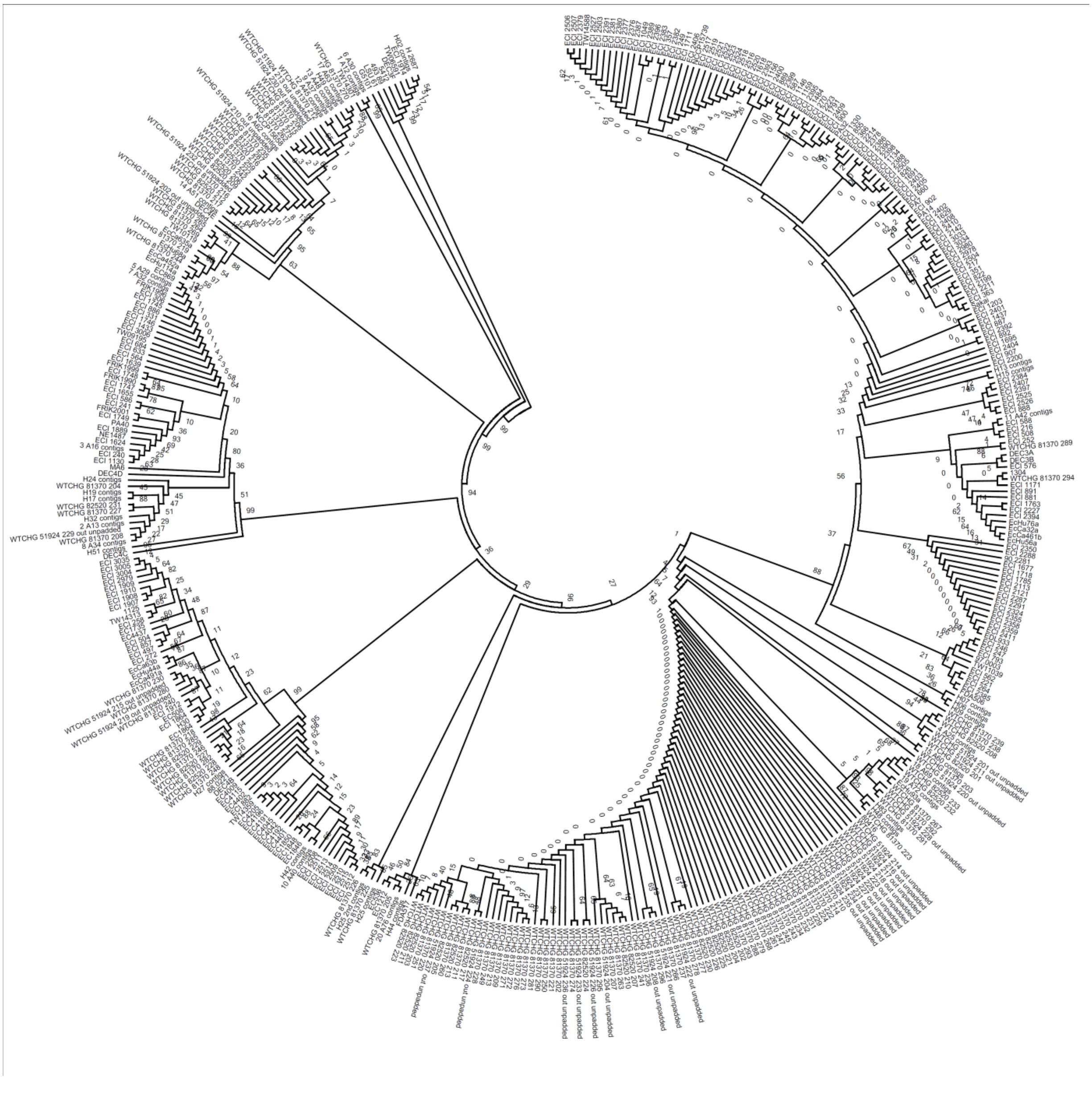

(b)

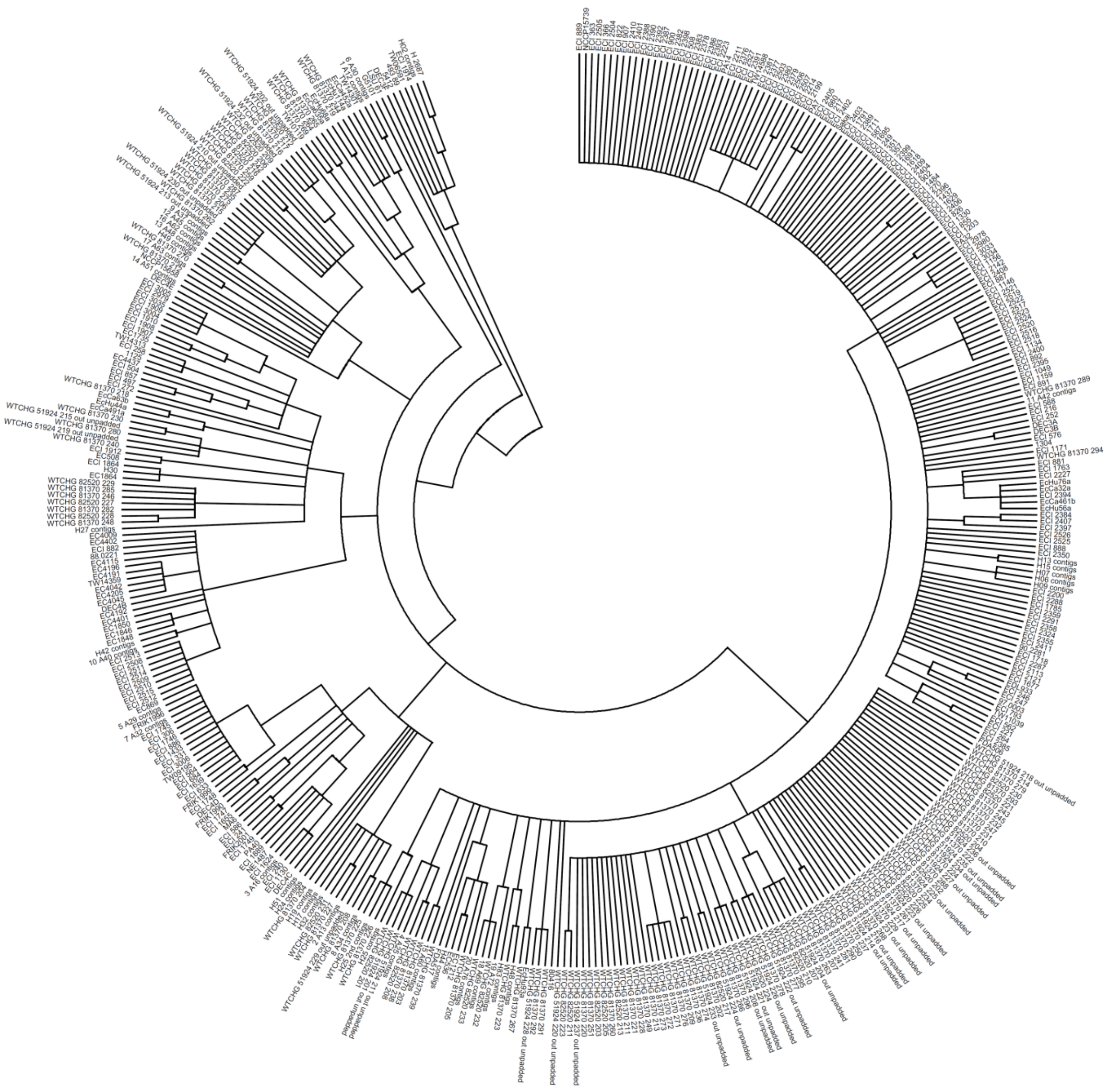

Fig. S3 (a) Bootstrapped phylogenetic tree and (b) the Consensus tree all of the isolates in the study.

(a) Sources: Clinical (●);Cattle (●);Sheep (●); Food/Environment (●); Unknown (●).

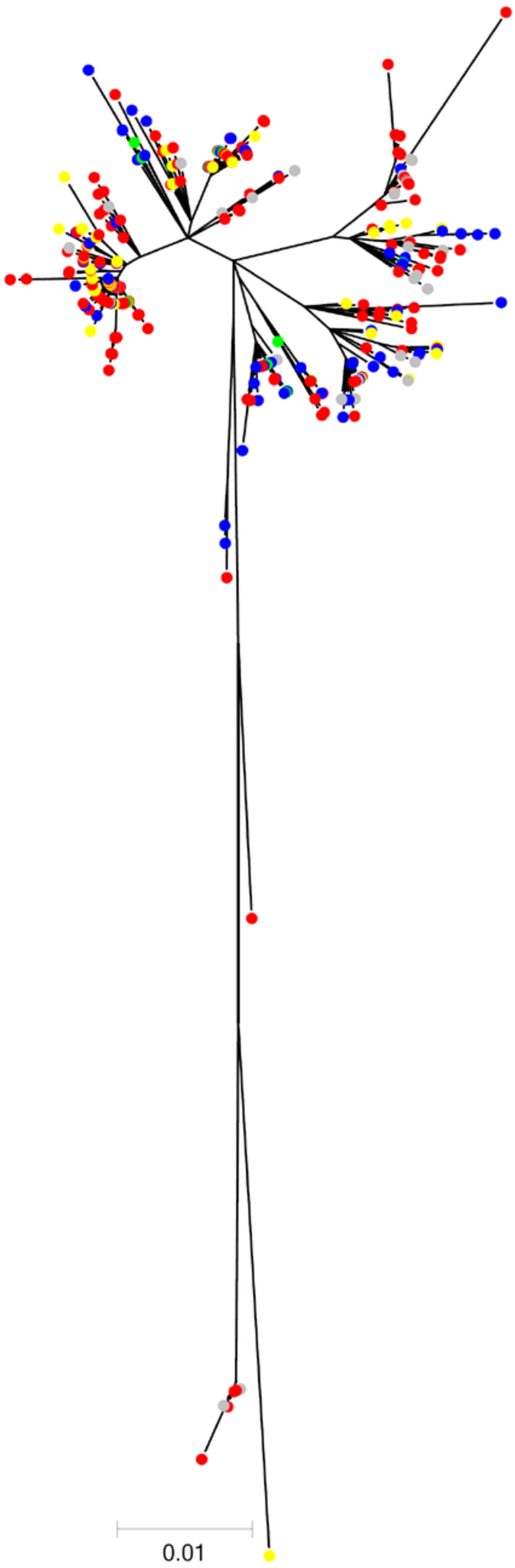

(b) Manning's Clades: Clade 1(●); Clade 2(●); Clade 3(●);Clade 4/5/6/7/9(●); Clade 8(●).

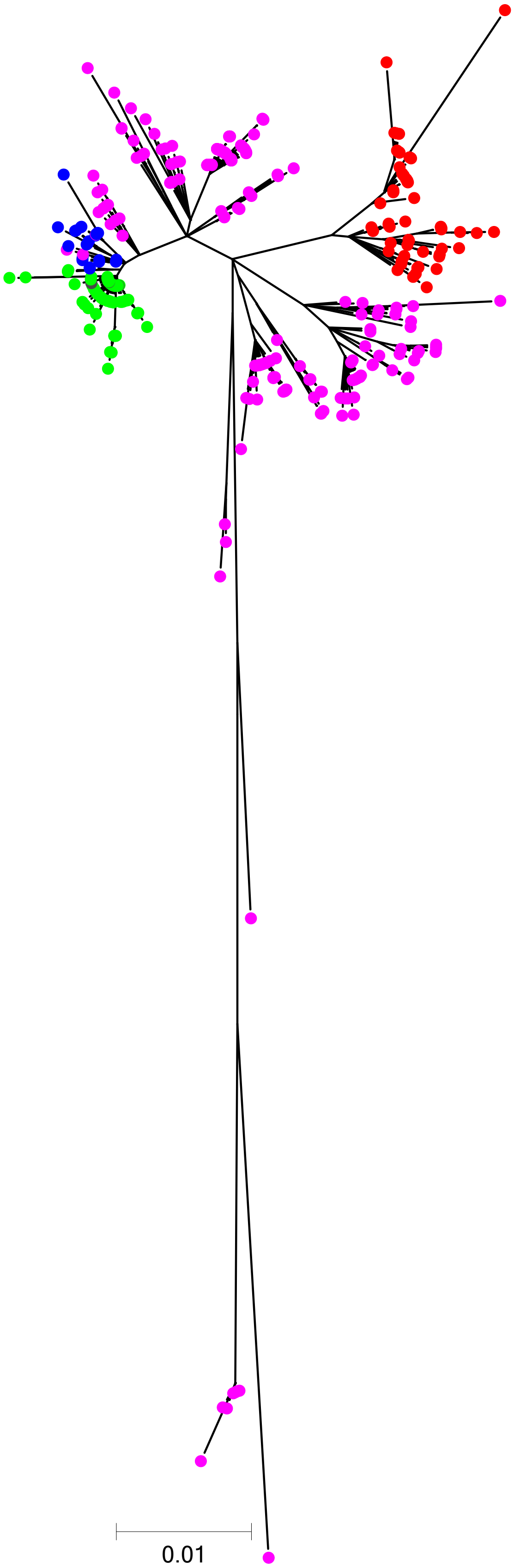

(c) LSPA6 lineages: I (●); I/II (●); II (●),

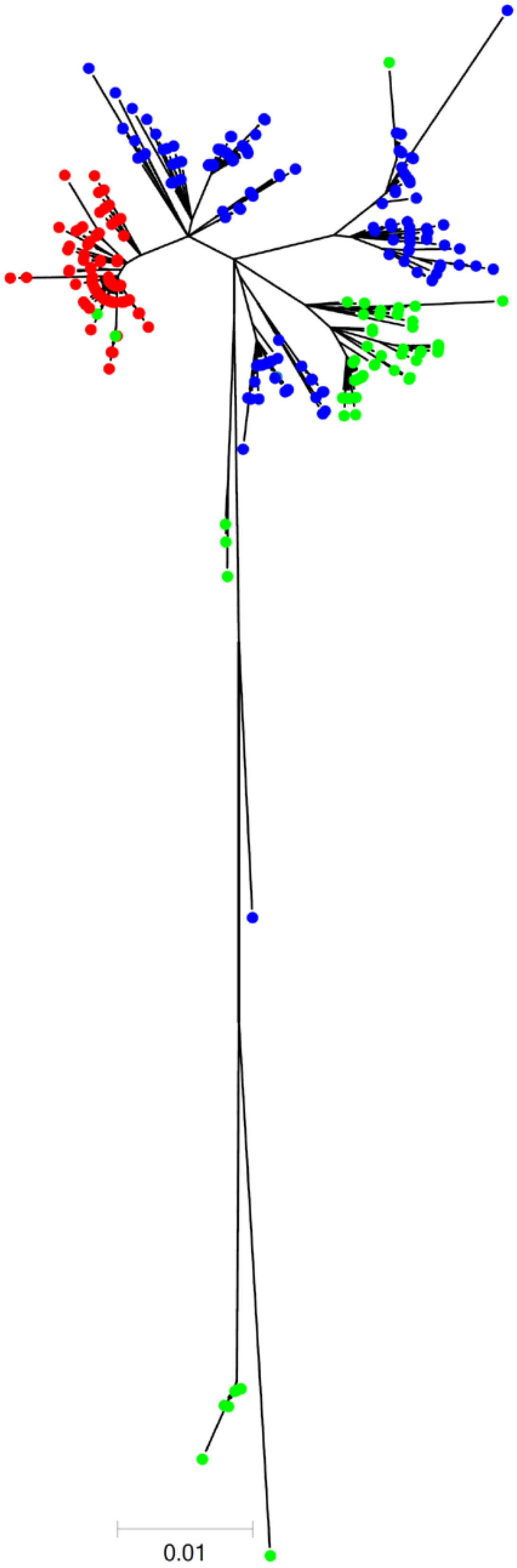

(d) *tir* 255 T (●); *tir* 255 A (●); Unknown (●);

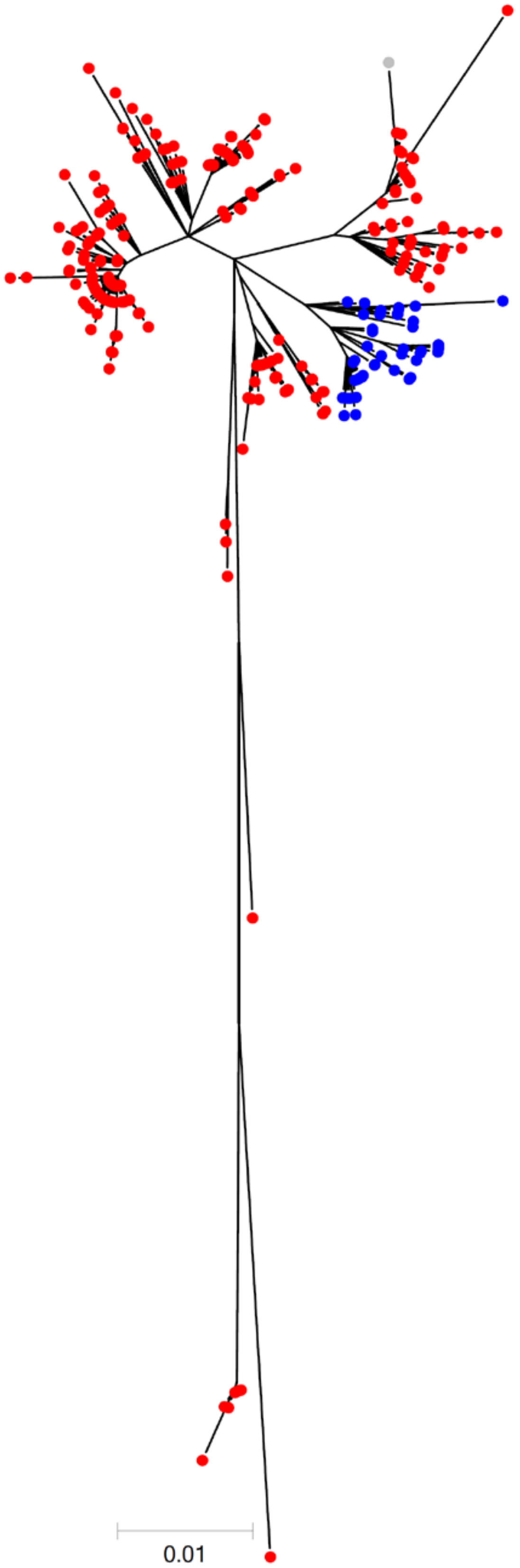

(e) Phage types: PT2 (●); PT21/28 (●); PT32 (●); PT8 (●); Other PTs (●); Unknown (○);

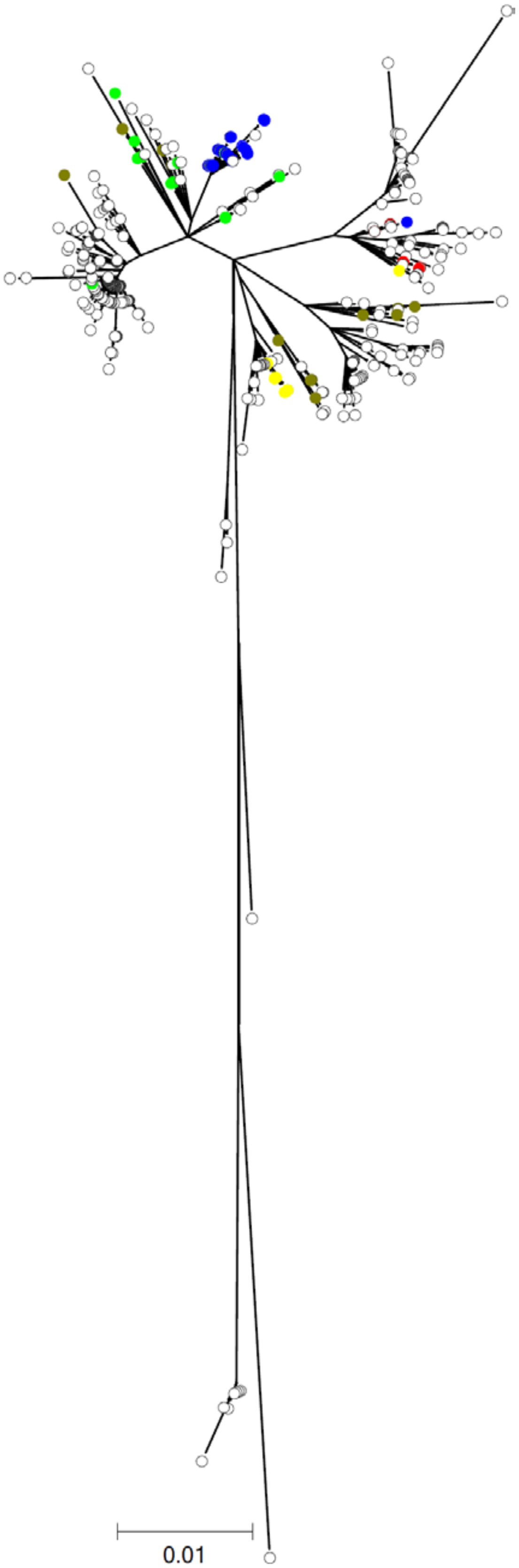

(f) Shiga toxins: *stx1a*(●); *stx1a/stx2a* (●); *stx1a/stx2c* (●); *stx2a* (●); *stx2a/stx2c* (●); *stx2c* (●); negative (●);*stx1a /stx2a/stx2c* (●),

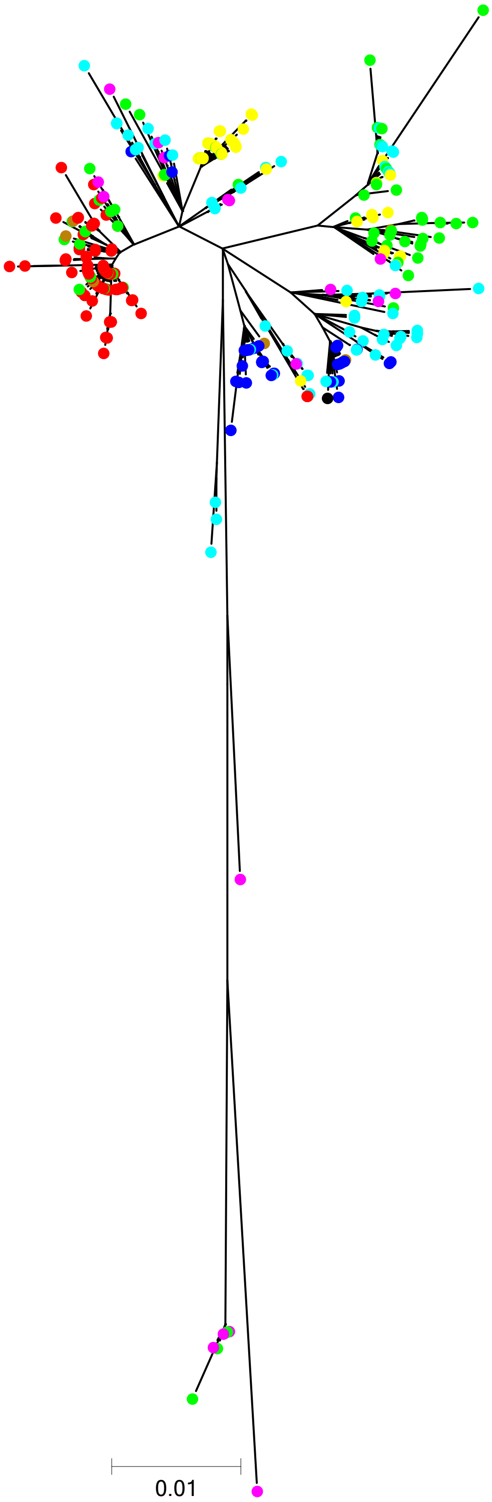

(g) SBI genotypes: WY1a2a (●), ASY2a2c (●); SY1a2c (●); SY2a2c (●); SY2c (●); AY2a (●);YN (●); Other SBI types (●); Unknown (●)

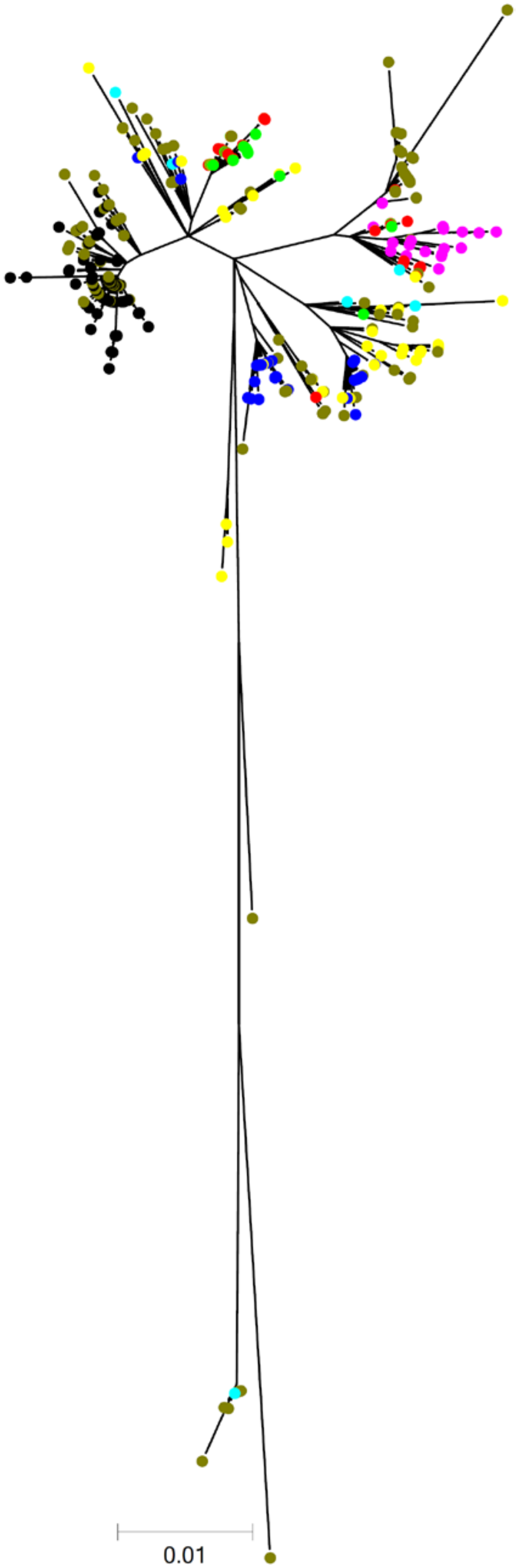

(h) *rfb* O157 positive (●) and negative (●).

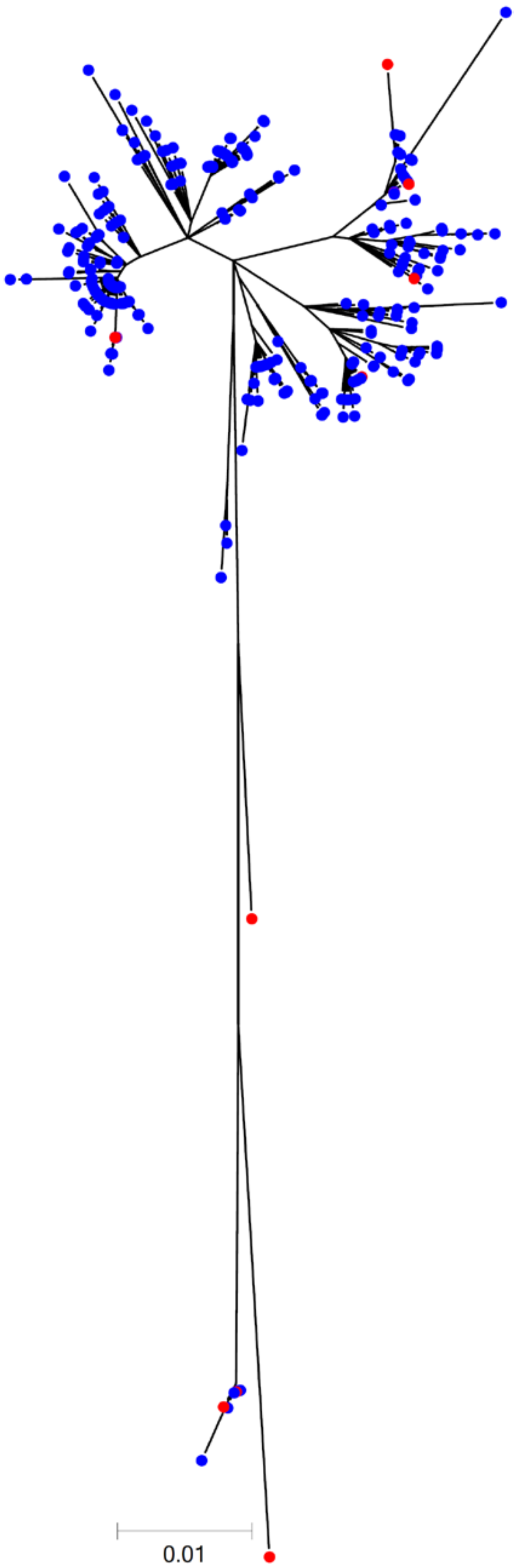

Fig. S4 International phylogeny by (a) Sources: Clinical (●);Cattle (●);Sheep (●); Food/Environment (●); Unknown (●),
(b) Manning's Clades: Clade 1(●); Clade 2(●); Clade 3(●);Clade 4/5/6/7/9(●); Clade 8(●),
(c) LSPA6 lineages: I (●); I/II (●); II (●),
(d) *tir* 255 T (●); *tir* 255A (●); Unknown (●);
(e) Phage types: PT2 (●); PT21/28 (●); PT32 (●); PT8 (●); Other PTs (●); Unknown (○);
(f) Shiga toxins: *stx1a*(●); *stx1a/stx2a* (●); *stx1a/stx2c* (●); *stx2a* (●); *stx2a/stx2c* (●); *stx2c* (●); *negative* (●);*stx1a /stx2a/stx2c* (●),
(g) SBI genotypes: WY1a2a (●), ASY2a2c (●); SY1a2c (●); SY2a2c (●); SY2c (●); AY2a (●);YN (●); Other SBI types (●); Unknown (●)
(h) *rfb* O157 positive (●) and negative (●).

Table S4 Oligonucleotide PCR primers, probes or gene accession numbers used in this study.

Target	Primer/Probe	Sequence 5’-3’ or gene accession no.	Amplicon size	Reference
rfb $_{O157}$ (O-antigen-encoding)	O157F<br>O157R	CGGACATCCATGTGATATGG<br>TTGCCTATGTACAGCTAATCC	259	[5]
eaeA (intimin)	eaeAF<br>eaeAR	GACCCGGCACAAGCATAAGC<br>CCACCTGCAGCAACAAGAGG	384	[5]
hlyA (plasmid-encoded enterohemolysin)	hlyAF<br>hlyAR	GCATCATCAAGCGTACGTTCC<br>AATGAGCCAAGCTGGTTAAGCT	534	[5]
stx genes	N/A		N/A	[6, 7]
stx1a		L04539.1		
stx1c		Z36901.1		
stx1d		AY170851.1		
stx2a		X07865.1		
stx2b		X65949.1		
stx2c		AB071845.1		
stx2d		AY095209.1		
stx2e		AJ249351.2		
stx2f		AB472687.1		
stx2g		AY286000.1		
[a]<u>LSPA6</u>				[8]
folD-sfmA	Forward<br>Reverse	TACGTAGGTCGAAGGG<br>CCAGATTTACAACGCC	161/170	
Z5935	Forward<br>Reverse	GTGTTCCCGGTATTTG<br>CTCACTGGCGTAACCT	133/142/160	
yhcG	Forward<br>Reverse	CTCTGCAAAAAACTTACGCC<br>CAGGTGGTTGATCAGCG	392/394/472	
rbsB	Forward<br>Reverse	AGTTTAATGTTCTTGCCAGCC<br>ATTCACCGCTTTTTCGCC	218	
rtcB	Forward<br>Reverse	GCGCCAGATCGATAAAGTAAG<br>GCCGTTGTAAACGTGATAAAG	270/279/288	
arp-iclR	Forward<br>Reverse	GCTCAATCTCATAATGCAGCC<br>CACGTATTACCGATGACCG	314/323	
[b]*tir* 255 SNP probes	255A<br>255T	ACTGAATGATGGATTTG<br>CTGAATGAAGGATTTG	NA<br>NA	[9]
[c]<u>Clade probes</u>				[10]
ECs2521	SNP3T<br>SNP3G	CAGTTACAC**T**CCAGCGATC<br>CAGTTACAC**G**CCAGCGATC	NA<br>NA	
ECs2357	SNP39A<br>SNP39C	CGCGCAATT**A**CGAGCCACT<br>CGCGCAATT**C**CGAGCCACT	NA<br>NA	
ECs3881	SNP24T<br>SNP24C	CGTGTGCAC**T**GGTTGCCGT<br>CGTGTGCAC**C**GGTTGCCGT	NA<br>NA	
ECs4130	SNP30T<br>SNP30C	TGCCGCTGG**T**GGCTTAAGC<br>TGCCGCTGG**C**GGCTTAAGC	NA<br>NA	
[d]<u>Bacteriophage insertion sites</u>				[11]
argW	Left junction	GCATCTCACCGACGATAACA<br>AATTAGCCCTTAGGAGGGGC	462	
	Right junction	CCGTAACGACATGAGCAACAAG<br>GCAGTATCACGCAGAGCTGAAG	583	
	Insertion site	CCGTAACGACATGAGCAACAAG<br>AATTAGCCCTTAGGAGGGGC	216	
sbcB	Left junction	GTGCTTGGGTCTTTTCTCTG<br>TCCAGGCGTAAGGATCGTAG	914	
	Right junction	GACAGCAGAAACAACGGATTTAAC<br>CCAGCGTGGGATAAAAGAGAATC	730	
	Insertion site	GACAGCAGAAACAACGGATTTAAC	406	

		TCCAGGCGTAAGGATCGTAG		
wrbA	Left junction	CCGACCTTTGTACGGATGTAA<br>CGAATCGCTACGGAATAGAGA	506/537	
	Right junction	AGGAAGGTACGCATTTGACC<br>ATCGTTCGCAAGAATCACAA	592	
	Insertion site	AGGAAGGTACGCATTTGACC<br>CGAATCGCTACGGAATAGAGA	314	
yehV	Left junction	CACCGGAAGGACAATTCATC<br>AACAGATGTGTGGTGAGTGTCTG	824	
	Right junction	AAGTGGCGTTGCTTTGTGAT<br>GATGCACAATAGGCACTACGC	702	
	Variant right jn.	AAGTGGCGTTGCTTTGTGAT<br>AGCGATACAGATCTCAACAC	295	
	Insertion site	AAGTGGCGTTGCTTTGTGAT<br>AACAGATGTGTGGTGAGTGTCTG	340	

[a]LSPA6 sub-typing results were arranged in the order *folD-sfmA*, *Z5935*, *yhcG*, *rbsB*, *rtcB* and *arp-iclR*. Each allele was assigned a number as described previously (Yang et al., 2004). Isolates showing the LSPA6 genotype 111111 were classified as LSPA6 lineage I (LSPA6 LI), while those with LSPA6 genotype 211111 were classified as LSPA6 lineage I/II (LSPA6 LI/II). Unique alleles (aberrant amplicon size) were assigned new numbers. All deviations from the genotypes 111111 and 211111 were classified as LSPA6 lineage II (LSPA6 LII).

[b]tir 255T>A polymorphism analysis was used to detect a single nucleotide polymorphism (A/T) located in the *tir* gene [9, 12].

[c]Clade Identification (Manning) utilised an in silico probe based method based on four SNPs [13] to identify the main Clades (1, 2, 3 and 8 independently and 4, 5, 6, 7 and 9 as a group) published previously [10].The probes were developed using the Sakai reference genome (BA000007.2).

[d]SBI results were concatenated using the terminology described previously [14] and restated here. When either or both bacteriophage-chromosome insertion site locus junctions were detected, the locus was considered occupied. When an intact locus insertion site product was detected without amplification of either bacteriophage insertion site junction, the locus was considered unoccupied. A modified genotyping code was assigned to each isolate using the characters A, S, W, Y, 1a, 2a and 2c to represent *argW*, *sbcB*, *wrbA*, *yehV*, *stx1a*, *stx2a* and *stx2c*, respectively. An N was assigned to any isolate that did not contain any of the three *stx* genes. Statistical differences in the frequencies of SBI types between animal host species and between countries were determined using the chi-square test.

Table S5. Abundance of LSPA6, *tir* 255, and Manning clades by source (Bovine, Ovine, Food/Environment and Clinical) for the Scottish *E. coli* O157 isolates.

		No. (%) of strains with factor				
Factor	Sub- type	Bovine (n=26)	Ovine (n=25)	Food/Env. (n=20)	Clinical (n=74)	Statistically significant pairwise comparisons[a]
LSPA6	Sub-type					
	111111 (I)	0 (0.0)	0 (0.0)	1 (5.0)	1 (1.4)	nd
	2N1121 (II)	0 (0.0)	0 (0.0)	0 (0.0)	1 (1.4)	nd
	252122 (II)	0 (0.0)	0 (0.0)	0 (0.0)	1 (1.4)	nd
	221132 (II)	0 (0.0)	0 (0.0)	0 (0.0)	1 (1.4)	nd
	221122 (II)	0 (0.0)	0 (0.0)	1 (5.0)	1 (1.4)	nd
	221111 (II)	0 (0.0)	0 (0.0)	0 (0.0)	1 (1.4)	nd
	211111 (I/II)	26(100.0)	25(100.0)	18 (90.0)	68 (90.0)	none
	Lineage					
	I	0 (0.0)	0 (0.0)	1 (5.0)	1 (1.4)	nd
	I/II	26(100.0)	25(100.0)	18 (90.0)	68 (91.9)	nd
	II	0 (0.0)	0 (0.0)	1 (5.0)	5 (6.7)	nd
tir 255	A allele	0 (0.0)	0 (0.0)	1 (5.0)	4 (5.4)	nd
Manning Clades	1	0 (0.0)	0 (0.0)	0 (0.0)	0 (0.0)	nd
	2	0 (0.0)	0 (0.0)	0 (0.0)	0 (0.0)	nd
	3	0 (0.0)	0 (0.0)	0 (0.0)	1 (1.4)	none
	4/5/6/7/9	25 (96.2)	24 (96.0)	25 (96.2)	68 (91.9)	CF, BF, FO
	8	1 (3.8)	1 (4.0)	1 (3.8)	5 (6.8)	CF, BF, FO

[a]Listed are statistically different pairwise comparisons where B = bovine, O = ovine, F = Food/Environmental and C = clinical. Statistical significance determined by a two tailed Fisher's exact test.

Table S6. Pairwise comparisons of Nei's genetic distances calculated using SNP polymorphisms from the PANSEQ analysis of the Scottish *E. coli* O157 isolates, between sources, phage types, Shiga toxin types, LSPA6 lineages and SBI genotypes.

Factor	Pairwise Comparison	Genetic Distance (d1)	*P*-value
Source:	Clinical-Cattle	0.00385	**0.006**
	Clinical-Sheep	0.00415	**0.007**
	Clinical-Food/Env.	0.00614	**0.0002**
	Cattle-Sheep	0.00278	0.0651
	Cattle-Food/Env.	0.00656	**<0.0001**
	Sheep-Food/Env.	0.00669	**0.0001**
Phage type:	PT21/28 - PT32	0.00668	**<0.0001**
	PT21/28 - PT8	0.00939	**<0.0001**
	PT21/28 - PT2	0.01389	**<0.0001**
	PT21/28 - Other	0.00993	**<0.0001**
	PT32 - PT8	0.01035	**<0.0001**
	PT32 - PT2	0.01495	**<0.0001**
	PT32 - Other	0.01001	**<0.0001**
	PT8 - PT2	0.01230	**<0.0001**
	PT8 - Other	0.01039	**<0.0001**
	PT2 - Other	0.01578	**<0.0001**
Shigatoxin type:	stx2a,2c - stx1a,2c	0.01015	**<0.0001**
	stx2a,2c - stx1a,2a	0.01096	**0.0008**
	stx2a,2c – stx2a	0.00349	0.0547
	stx2a,2c - stx2c	0.01496	0.0691
	stx2a,2c - stx-ve	0.00908	**0.0001**
	stx1a,2c - stx1a,2a	0.00984	**0.0001**
	stx1a,2c – stx2a	0.00946	**<0.0001**
	stx1a,2c - stx2c	0.00978	**<0.0001**
	stx1a,2c - stx-ve	0.01082	**<0.0001**
	stx1a,2a – stx2a	0.01032	**0.0003**
	stx1a,2a - stx2c	0.01032	**0.0082**
	stx1a,2a – stx-ve	0.01128	**0.0037**
	stx2a – stx2c	0.01008	**<0.0001**
	st2a - stx-ve	0.00949	**<0.0001**
	stx2c - stx-ve	0.00881	**0.008**
LSPA6 lineage	LSPA I/II - LSPA II	0.01404	**<0.0001**
SBI genotypes	ASY2a2c - SY1a2c	0.01012	**<0.0001**
	ASY2a2c - SY2a2c	0.00277	**0.0202**
	ASY2a2c - SY2c	0.01065	**0.0001**
	ASY2a2c - YN	0.00856	**0.0204**
	ASY2a2c - Other	0.00680	**<0.0001**
	SY1a2c - SY2a2c	0.01016	**<0.0001**
	SY1a2c - SY2c	0.01094	**<0.0001**
	SY1a2c - YN	0.01113	**<0.0001**
	SY1a2c - Other	0.00862	**0.0001**
	SY2a2c - SY2c	0.01011	**<0.0001**
	SY2a2c - YN	0.00880	**0.0153**
	SY2a2c - Other	0.00644	**<0.0001**
	SY2c - YN	0.01031	**0.0071**
	SY2c - Other	0.00934	**0.0011**
	YN - Other	0.00905	**0.0193**

Table S7. SBI typing P-values for pairwise comparisons of (a) rarefaction and (b) Simpson's index by (i) animal source and (b) country.

(a) Rarefaction

(i) Animal Source

	Cattle	Sheep
Cattle	-	0.091
Sheep	-	-

(ii) Country

	USA	Canada	Netherlands	New Zealand	Scotland
USA	-	0.137	0.630	0.149	0.363
Canada	-	-	0.146	0.513	0.055
Netherlands	-	-	-	0.158	0.410
New Zealand	-	-	-	-	
Scotland	-	-	-	-	-

(b) Simpson's index

(i) Animal Source

	Cattle	Sheep
Cattle	-	0.356
Sheep	-	-

(ii) Country

	USA	Canada	Netherlands	New Zealand	Scotland
USA	-	**<0.0001**	0.557	**0.038**	0.125
Canada	-	-	**<0.0001**	**0.026**	**<0.0001**
Netherlands	-	-	-	**0.017**	0.071
New Zealand	-	-	-	-	0.146
Scotland	-	-	-	-	-

Table S8 Relationship between phylogenetic clades (Fig. 4) and previous typing methods[a].

Clades current paper	Clades [10]	LSPA6 lineages [8]	*tir* 255 [9, 10]	Phage Types[e,f]	Shigatoxins[e]	SBI types[e]
A	4/5/6/7/9	II[b]	T		-ve, *stx2a*	Other
B	4/5/6/7/9	II	T		*stx2c*	SY2c
C	4/5/6/7/9	I/II	T	PT8	*stx1a/stx2c*	SY1a2c & Other
D	4/5/6/7/9	I/II	T	"Other" PTs	*stx2c*	Other
E (i)	4/5/6/7/9	II	A		*stx2c, stx1a/stx2c*	SY2c, SY1a2c & other
E (ii)	4/5/6/7/9	II	A	"Other" PTs	*stx2c*, -ve	Other
F (i)	8	I/II	T	PT2	*stx2a*	AY2a, ASY2a2c
F (ii)	8	I/II[c]	T		*stx2a,stx2c*	Other
G (i)	4/5/6/7/9	I/II	T	PT32	*stx2c*	SY2c, Other
G (ii)	4/5/6/7/9	I/II	T	PT21/28	*stx2a/stx2c*	ASY2a2c, SY2a2c
G (iii)	4/5/6/7/9	I/II	T	PT32	*stx2a, stx2c,* -ve	Other
G (iv)	4/5/6/7/9	I/II	T	PT32	*stx2c*	SY2c, Other
G (v)	4/5/6/7/9	I	T		*stx2a*, -ve	Other, WY1a2a
G (vi)	1, 2, 3 & 4/5/6/7/9	I[d]	T		*stx1a/stx2a, stx2a*	WY1a2a. Other

[a]Phylogenies annotated for each of the typing methods are given in Suppl. Fig. S4.
[b]One isolate lineage I/II,
[c]One isolate lineage II,
[d]Two isolates lineage II,
[e]Predominant types in the clade
[f]Phage typing was only available for the majority of Scottish isolates and EDL933 and Sakai.

Table S9 Pairwise comparisons of Nei's genetic distances calculated using SNP polymorphisms obtained from the PANSEQ analysis of all Scottish and international *E. coli* O157 isolates between and within countries[a].

Comparisons	Countries	Genetic Distance(d1)	P-value
Comparing ***all*** isolates between countries	USA - Canada	0.01538	**0.0001**
	USA - Netherlands	0.01679	**0.0014**
	USA - New Zealand	0.01767	**0.0003**
	USA - Scotland	0.01998	**<0.0001**
	Canada - Netherlands	0.01707	**<0.0001**
	Canada - New Zealand	0.01496	**0.0016**
	Canada - Scotland	0.01487	**<0.0001**
	Netherlands - New Zealand	0.01971	**0.0005**
	Netherlands - Scotland	0.01672	**<0.0001**
	New Zealand - Scotland	0.01541	**<0.0001**
Comparing ***clinical*** isolates between countries	USA - Canada	0.01518	**0.0039**
	USA - Netherlands	0.01860	**0.0027**
	USA - New Zealand	0.01829	**0.0361**
	USA - Scotland	0.01998	**<0.0001**
	Canada - Netherlands	0.01746	**0.0002**
	Canada - New Zealand	0.01549	**0.0368**
	Canada - Scotland	0.01581	**<0.0001**
	Netherlands - New Zealand	0.02023	0.1062
	Netherlands - Scotland	0.01868	**<0.0001**
	New Zealand - Scotland	0.01568	**0.0015**
Comparing ***cattle*** isolates between countries	USA - Canada	0.02003	**0.0024**
	USA - Netherlands	0.02158	**0.0015**
	USA - New Zealand	0.02460	**0.0001**
	USA - Scotland	0.02351	**0.0001**
	Canada - Netherlands	0.01686	**<0.0001**
	Canada - New Zealand	0.01418	**0.0093**
	Canada - Scotland	0.01761	**<0.0001**
	Netherlands - New Zealand	0.02009	**0.0007**
	Netherlands - Scotland	0.01702	**<0.0001**
	New Zealand - Scotland	0.01749	**0.0002**
Comparing ***clinical*** and ***cattle*** isolates within country	Scotland	0.00633	**0.0071**
	Canada	0.01068	**0.0014**
	Netherlands	0.01930	**<0.0001**
	New Zealand	0.00595	0.5059[*]
	USA	0.02550	**0.0079**

[a]The number of isolates for each country are Canada (87 clinical & 23 cattle), Netherlands (19 clinical and 20 cattle), Scotland (74 cclinical and 26 cattle), New Zealand (6 clinical and 6 cattle), USA (15 clinical and 4 cattle).
